# Lived experiences, depression, and quality of life in Korean women with Parkinson disease after deep brain stimulation: a multimethod study

**DOI:** 10.4069/whn.2025.08.29

**Published:** 2025-09-30

**Authors:** Mi Sun Kim, Sun Ju Chung

**Affiliations:** 1 Departemth of Nursing, The Graduate School of Chung-Ang University, Seoul, Korea; 2 Department of Nursing, Kyungmin University, Uijeongbu, Korea; 3 Department of Neurology, Asan Medical Center, University of Ulsan College of Medicine, Seoul, Korea

**Keywords:** Deep brain stimulation, Depression, Parkinson disease, Quality of life

## Abstract

**Purpose:**

Although deep brain stimulation (DBS) is increasingly used to manage Parkinson disease (PD), its effects on women’s experiences and quality of life (QoL) remain underexplored. This study examines the experiences of women with PD after DBS implantation and identifies changes in QoL and depression, integrating qualitative and quantitative findings to investigate sex-specific treatment outcomes.

**Methods:**

A multimethod design was employed, beginning with in-depth interviews and followed by quantitative assessments using the Unified Parkinson’s Disease Rating Scale (UPDRS), Beck Depression Inventory (BDI), and the Parkinson’s Disease Questionnaire-39 (PDQ-39) before and after DBS. Data were collected between 2019 and 2021, and findings were integrated to provide a comprehensive understanding of the effects of DBS.

**Results:**

Twelve women with PD who underwent DBS implantation participated. Qualitative analysis revealed themes including “stress due to external changes observed by others,” “pain at the implantation site,” “decline in DBS effectiveness,” “economic burden of battery replacement surgery,” and “anxiety when DBS is turned off.” Quantitative analysis showed significant improvement in UPDRS part III scores following surgery (39.32±10.40 vs. 25.01±16.82, *p*=.038). However, no significant differences were found in BDI and PDQ-39 scores before and after surgery (*p*-values ranged from .450 to .695). Integrating both datasets highlighted the interplay of physical improvement with persistent psychosocial challenges.

**Conclusion:**

Although DBS improved motor symptoms, women with PD continued to experience emotional and financial distress. These findings underscore the importance of holistic, patient-centered care that addresses both medical and psychosocial needs.

## Introduction

Parkinson disease (PD) is a neurodegenerative disorder characterized by the loss of dopaminergic neurons in the midbrain. According to National Health Insurance Corporation data, approximately 171 per 100,000 individuals had PD between 2011 and 2015 in South Korea (hereafter, Korea) [1]. In Western regions such as North and South America and Europe, PD is more prevalent in men than women [2]. However, in Asian countries, including Korea, the prevalence is higher among women [3,4].

Treatment for PD includes both drug therapy and surgical options such as deep brain stimulation (DBS). DBS implantation alleviates motor fluctuations and dyskinesia that remain uncontrolled despite optimal medication [5], and it helps reduce drug-induced side effects [6]. According to statistics from the Health Insurance Review and Assessment Service, 1,968 patients underwent DBS implantation in Korea between 2010 and 2019. Globally, more than 100,000 patients have undergone DBS, and this number is expected to continue rising [7]. Previous studies in Western countries have reported both the clinical effects of DBS and patients’ experiences; however, most participants were men, and only a limited number of studies have addressed women [8]. Moreover, no studies to date have examined the quality of life (QoL) of women with PD who have undergone DBS in Asia, including Korea.

Because the timing of DBS surgery for women often coincides with menopause, these patients experience multiple physical and emotional changes [9], making this factor especially relevant. Therefore, this study aimed to examine improvements in QoL, depression, and the lived experiences of Korean women with PD following DBS implantation.

## Methods

**Ethics statement**

This study was approved by the Institutional Review Board of Asan Medical Center (ASAN IRB No. 2019-0903). All participants provided written informed consent after receiving a full explanation of the study and were informed that they could withdraw their consent at any time during the study. The authors affirm that they have read the Journal’s position on issues related to ethical publication and confirm that this work complies with those guidelines.

### Participants selection

This study included 12 women with PD who underwent DBS implantation between March 2002 and September 2019 in the Department of Neurosurgery. Women with PD who had received DBS were enrolled and examined at the Department of Neurology of a tertiary hospital between December 2019 and April 2020. The inclusion criteria were: (1) age ≥18 years and (2) sufficient cognitive ability to complete questionnaires and interviews. The exclusion criteria were: (1) diagnosis of dementia due to disease progression and (2) presence of cancer, stroke, or renal failure that could introduce uncertainty into disease outcomes. Participants were recruited through purposive sampling. Each patient received a thorough explanation of the study’s objectives, methods, and their rights as participants. Informed consent was obtained voluntarily, with all participants signing written consent forms.

### Study design and data collection

This study employed a multimethod approach [10,11], integrating qualitative and quantitative data to examine the experiences of women with PD after DBS implantation and to assess changes in QoL and depression. A qualitative exploratory study was first conducted to provide in-depth insights and contextual understanding of the quantitative results. Semi-structured, open-ended interviews with female PD patients who had undergone DBS allowed detailed exploration of their lived experiences, including sources of satisfaction and dissatisfaction. These findings guided the subsequent quantitative analysis, which evaluated motor symptoms, QoL, and depression using validated scales. The study adhered to COREQ (Consolidated Criteria for Reporting Qualitative Research) guidelines (https://www.equator-network.org/reporting-guidelines/coreq/) and the STROBE (Strengthening the Reporting of Observational Studies in Epidemiology) statement (https://www.strobe-statement.org/).

### Qualitative methods

We conducted semi-structured interviews with women with PD who had undergone DBS implantation to explore their individual life experiences and satisfaction or dissatisfaction. Each patient participated in three interview sessions, each lasting 1 to 2 hours, during outpatient visits, and all interviews were recorded. Additional data were collected through follow-up questions via phone calls. Qualitative data were collected by asking questions to confirm the experiences of PD patients after DBS implantation. The questions were as follows:

• What are the lived experiences of individuals following DBS implantation?

• After DBS surgery, what have you been satisfied with?

• What have you been dissatisfied with while living with DBS?

The interview data were analyzed using a thematic analysis approach [12]. The four evaluation criteria for qualitative research proposed by Guba and Lincoln [13] in 1981—credibility, transferability, dependability, and confirmability—were applied. To strengthen credibility, the study was conducted rigorously, adhering to established qualitative methodologies under expert guidance. Conceptualization and categorization were performed after data analysis, and these processes underwent three rounds of review by three experienced nursing scholars to ensure validity and appropriateness. The principal investigator, who has worked as a neurology clinical nurse specialist with a focus on PD care for 18 years (from July 1, 2005 to September 30, 2023), contributed expertise to the study. This prolonged engagement and sustained observation further enhanced credibility. Transferability was supported through the systematic collection and documentation of detailed, context-rich data. All interviews were recorded and organized to create a comprehensive dataset, thereby facilitating the application of findings to diverse contexts. Dependability, reflecting the consistency and reproducibility of the results, was upheld through transparent documentation, including audio recordings of all interviews and systematic descriptions of coding and categorization. Finally, confirmability was established through triangulation involving nursing experts, study participants, and members of the general public who were not nursing professionals. This multilayered validation process confirmed the impartiality and rigor of the study’s findings.

### Quantitative methods

QoL, depression levels, and demographic characteristics were retrospectively obtained from the electronic medical record of the neurology department at a tertiary hospital between April 2020 and December 2021.

• QoL: The Parkinson Disease Questionnaire-39 (PDQ-39) [14], and its Korean version [15], was administered both before and after DBS implantation to assess QoL. The PDQ-39 is a disease-specific instrument for PD, divided into eight sections: mobility (10 items), activities of daily living (ADL, six items), emotional well-being (six items), stigma (four items), social support (three items), cognition (four items), communication (three items), and bodily discomfort (three items). Each item is scored on a 5-point Likert scale (0, never to 4, always). Lower converted scores (range, 0–100) indicate better disease-specific QoL.

• Depression: Depression was measured using the 21-item Korean version [16] of the Beck Depression Inventory (BDI) [17]. Items are scored from 0 (never) to 3 (severe), yielding a total score between 0 and 63, with higher scores indicating greater severity.

• Demographic and clinical characteristics: Variables collected included age, marital status, age at onset, age at diagnosis, disease duration, age at DBS implantation, duration since implantation, surgical site, occupation, comorbidities, prolonged use of selective serotonin reuptake inhibitors (SSRIs), axial symptoms, motor fluctuations, dyskinesia, off duration, presence of impulse control disorders, levodopa equivalent daily dose (LED), monthly income, and levodopa responsiveness before and after surgery. Disease severity and progression were assessed using the Unified Parkinson’s Disease Rating Scale (UPDRS) and the modified Hoehn and Yahr (H&Y) stage. The UPDRS is a standardized clinical tool composed of four parts: Part I evaluates mentation, behavior, and mood; Part II assesses ADL; Part III measures motor function; and Part IV examines treatment-related motor and non-motor complications [18]. The H&Y stage is a widely used ordinal scale that categorizes PD progression into five stages, with higher stages representing more severe motor impairment and functional decline [19].

Data were analyzed using IBM SPSS ver. 21.0 for Windows (IBM Corp., Armonk, NY, USA). Statistical significance was set at *p*<.05. To compare baseline and post-DBS outcomes, motor and non-motor symptoms, medication side effects, QoL, and BDI were evaluated. Continuous variables were analyzed with the Wilcoxon signed-rank test, and categorical variables with the McNemar test. Patients with improved QoL after DBS were compared with those without improvement. The Mann-Whitney U-test was used for continuous variables, and the chi-square test for categorical variables.

## Results

### Demographic and clinical characteristics

The mean age of the 12 participants was 57.50 years (range, 40–67 years). The mean age at DBS implantation was 51.42 years (range, 37–64 years), and the mean disease duration was 17.01 years (range, 11.3–30.4 years). The average duration since DBS implantation was 5.92±4.87 years (range, 1–18 years) (Tables 1, 2).

### In-depth interview findings after deep brain stimulation implantation

Five theme clusters captured the satisfactory and dissatisfactory experiences of women with PD following DBS implantation (Table 3): (1) stress due to external changes seen by others, (2) pain at the DBS implantation site, (3) reduction in DBS effectiveness, (4) economic burden of battery replacement surgery, and (5) anxiety when DBS turns off.

#### • Theme cluster 1. Stress due to external changes seen by others

Women with PD who underwent DBS implantation reported significant distress related to visible surgical changes. These alterations caused discomfort, embarrassment, and heightened self-consciousness in social and public settings, influencing both daily life and self-perception.

##### Theme 1.1. Feelings of anxiety and embarrassment in social settings

Participants described discomfort when others noticed their surgical sites, particularly in hair salons, swimming pools, and other public places. Reactions from hairdressers or strangers often intensified their embarrassment and self-consciousness, leading some to avoid public spaces altogether.

*“It’s sticking out here. (touching the surgical area on the head) When I go to a beauty salon, I am asked, “What is this?” I say that it is just because of the operation, but there are two horns on each side.” (Participant 1)*

*“When I go to the hair salon to dye my hair, I get a service to wash my hair. Whenever the hairdressers move my hair, they get confused and surprised, saying, “Oh my God.” I am embarrassed every time I go to the beauty salon. I explain, but it is uncomfortable to do so each time.” (Participant 6)*

*“I have never been to a public pool since I do not want others to see the surgical site. I am afraid I will be asked (touches her chest) what’s protruding, so I guess...... I am afraid to think of it as a strange robot, so I never go to the public pool.” (Participant 11)*

##### Theme 1.2. Emotional discomfort and self-perception

Participants expressed distress when observing surgical modifications on their bodies, especially during self-care activities such as showering. Many also changed their clothing styles to conceal scars or protrusions, which limited personal expression and reduced self-confidence.

*“Every time I take a shower, I can feel the left side (touching the area where the battery is inserted). ‘This protruding part must be the battery.’ At some point, I started to feel bad when I touched it. I also worried that people might stare at me. For a moment, I felt hurt in my heart.” (Participant 3)*

*“I used to wear sleeveless tops frequently because my shoulders and chest area were slim. However, I can no longer wear such clothing since the surgical site on my chest protrudes and the scar is visible.” (Participant 10)*

This theme highlights how DBS implantation, while functionally beneficial, introduces emotional and social challenges. It underscores the importance of holistic patient support, including body image counseling and social reintegration programs.

#### • Theme cluster 2. Pain at the deep brain stimulation implantation site

Persistent pain and discomfort at the DBS implantation site were commonly reported, particularly in the chest and head, where the battery and wires were located.

##### Theme 2.1. Numbness and dull pain

Some participants reported numbness and tingling at the surgical site, making daily activities such as showering or lying down uncomfortable.

*“What is difficult is that sometimes I feel tingling when I lie on my side or when taking a shower (pointing to the chest area).” (Participant 4)*

*“My chest area hurts and tingles when I charge the battery (touching the chest area where the battery is inserted). My head is numb and painful in the operated area. The senses are dull in there.” (Participant 9)*

##### Theme 2.2. Pain aggravated by certain postures

Pain was exacerbated by particular postures, physical activity, and intimate interactions, significantly affecting QoL and overall well-being.

*“When I’m in the shower, or when I’m putting my shoulder on and taking it off, or when I’m lying down, it’s very painful in my chest where the battery is inserted, but I’m thinking, ‘It’ll be okay after a while.’” (Participant 4)*

*“There has been a protrusion on the upper part of my chest, so I feel uncomfortable when lying on my side or exercising. My chest is a little stuffy when I am intimate with my partner. The device has made me uncomfortable since its implantation.” (Participant 10)*

##### Theme 2.3. Continuous awareness of the device

Participants reported constant awareness of the implanted device, with discomfort caused by wires and protrusions under the skin.

*“It started hurting around last autumn. (touching the implanted battery site on her chest) At first, I thought it was my heart. But then I realized that the area where the battery was implanted kept throbbing. That’s when I knew—it was because of the battery.” (Participant 3)*

*“I always feel like there’s something inside my body. There are wires in my neck, and when they get pressed, it really hurts. It’s very uncomfortable.” (Participant 6)*

This theme highlights the need for improved postoperative pain management, better device design, and sustained long-term patient support to improve QoL.

#### • Theme cluster 3. Reduction in deep brain stimulation effectiveness

Over time, some participants perceived a decline in the benefits of DBS. Although the initial effects of surgery were often positive, the progressive course of PD contributed to a gradual loss of symptom control, leading to pain, frustration, and emotional distress.

##### Theme 3.1. Gradual decline in symptom control

Initially, participants reported substantial relief, but symptoms such as freezing of gait, muscle weakness, and reduced motor control worsened over time.

*“Before surgery, my symptoms were limited to neck stiffness and toe curling. However, as time passed, I began experiencing severe freezing of gait, leading to frequent falls and injuries. Despite maintaining the same device settings, my walking ability had deteriorated, negatively affecting my quality of life.” (Participant 1)*

*“It has been more than 3 years since the operation and its effects are wearing off now. (After surgery) It was okay at first. The effect of the medicine was initially strong, but its effect has been decreasing with time. It is very difficult these days.” (Participant 4)*

*“The tremors have improved, but the muscle weakness still remains. I also feel like the device is less effective now. Twice a day, at around 2 PM and 5 PM, I suddenly lose all my strength. When that happens, I feel terrible. I can’t do anything—I just lie down.” (Participant 8)*

##### Theme 3.2. Psychological and emotional toll

As DBS effectiveness diminished, participants described frustration, emotional exhaustion, and in some cases hopelessness.

*“Even after the surgery, my body remains hunched forward, and I often need someone to support me from behind to keep me upright. Several times, the elevator doors opened, but I couldn’t lift my feet to step in. Now, because of that fear, I avoid elevators and deliberately use the stairs instead.” (Participant 2)*

*“After my first surgery, I found vitality in my life. I was able to work again, and I had hope. However, these days it’s harder. Recently, my body has been rolling on the floor due to severe dyskinesia, like a cerebral palsy patient shaking wildly. My body feels like a squid being grilled in a fire. I would rather die.” (Participant 11)*

##### Theme 3.3. Challenges in maintaining functionality

Participants struggled to balance device settings to achieve symptom relief without triggering new complications such as speech difficulties or involuntary movements.

*“Now, even when I adjust the device, my speech articulation doesn’t improve. The biggest issue for me is that I can’t speak properly. I tried increasing the device settings to improve my speech, but then my body started moving too much, so I had to come back to lower it again. When I think about my current condition, I wonder if I should have had the surgery at all.” (Participant 5)*

*“I used to be a very active person. But these days, when I go out, I struggle to move properly, and that makes me feel emotionally drained. Because of that, my social interactions have become limited. In those moments, I try increasing the device settings to help me move better. It does improve things a little, but even that isn’t easy.” (Participant 10)*

This theme emphasizes the importance of continuous clinical monitoring, personalized adjustments of device settings, and psychological support for long-term disease management.

#### • Theme cluster 4. Economic burden of battery replacement surgery

Participants frequently reported financial distress related to the recurring costs of battery replacement for DBS devices.

##### Theme 4.1. Anxiety over future costs

Patients, particularly those who were unemployed or homemakers, worried about affording future battery replacement operations, which led to emotional distress.

*“It’s going to cost me money every time I have to replace the battery, and I’m worried about the financial burden if I keep thinking about replacing the battery while this disease keeps going.” (Participant 6)*

*“I do not have enough money to exchange the batteries. Since I do not earn money, I am worried about covering this expense. If I do not have the money to exchange the battery later, I will just have to die.” (Participant 9)*

##### Theme 4.2. Cumulative financial burden

The recurring expenses of surgery, hospitalization, and caregiver assistance placed a heavy strain on families.

*“In the future, if the disease continues and there is no money, the cost of replacing the battery will be a burden to cover. Every time I change the battery, it costs a lot. I am concerned about where I can get financial assistance. Otherwise, I will have no choice but to die.” (Participant 6)*

*“I am concerned that the battery is now running out. I don’t know how many times it will work in the future, but I am worried about everything. Being hospitalized and undergoing surgery is a burden in itself.” (Participant 12)*

##### Theme 4.3. Dependence on financial support

Many participants expressed concern about burdening family members, leading to guilt and feelings of dependency.

*“My husband works hard, and we have no financial flexibility. We are not prepared for these costs. If I need to be hospitalized for a battery replacement, I would have to hire a caregiver, and a week-long stay would be too expensive.” (Participant 2)*

*“The disease will continue to progress, and I will have to keep replacing the battery. How will I afford it? I may need financial support when the time comes.” (Participant 5)*

*“The surgery costs so much money. And since the battery needs to be replaced every 4 to 5 years, I feel like I’m just burdening my husband and children. At this age, where would I even get the money...” (Participant 7)*

This theme highlights the urgent need for policy interventions and financial assistance programs to alleviate the economic burden of long-term DBS therapy.

#### • Theme cluster 5. Anxiety when deep brain stimulation turns off

For participants who underwent DBS implantation, the device became indispensable to daily functioning. However, the prospect of turning it off, even briefly, triggered profound anxiety and distress. Many expressed uncertainty about how their bodies would respond when the device was inactive, leading them to avoid medical procedures or situations requiring DBS deactivation.

##### Theme 5.1. Avoidance of medical procedures requiring deep brain stimulation deactivation

Some participants hesitated to undergo necessary treatments because of their fear of deactivating the DBS device. Concerns included how their bodies might react and whether they could successfully reactivate the device afterward.

*“I went to the dentist for treatment, but the dentist said they couldn’t do it because of my DBS. They told me to get a referral letter from the hospital where I usually go. I learned how to turn off the device, but I was so scared. What will happen to my body if the device turns off? What if I can’t turn it back on?” (Participant 1)*

*“I need a health check-up, but I can’t do it because I don’t know what to do with the machine (in the body) or what will happen.” (Participant 3)*

*“When I went to an oriental medicine clinic for treatment, they wanted to turn off my device before using a medical machine. But I was afraid it might break, so I refused the treatment. To treat my knee, they needed to use electrical therapy, but since I have a neurostimulator inside me, I had no choice. I told them not to use any electric treatment because I was too scared to turn off my device.” (Participant 7)*

##### Theme 5.2. Physical and emotional reactions to deep brain stimulation deactivation

Beyond uncertainty, some participants experienced severe physical and emotional symptoms when the device was turned off. The abrupt loss of mobility, combined with sensations of suffocation, often led to panic episodes and reinforced reliance on DBS.

*“I used to love getting massages in the sauna chair. But now, I can’t do it anymore… I’m too scared.” (Participant 8)*

*“If you turn off the machine, it hardens like a piece of wood, and I cannot move. I have been reliant on this machine. Once, when I went for an electrocardiogram, I turned off the machine beforehand. While I was waiting for the test with the DBS turned off, I had a panic episode, I could not move, and I ran out of breath. So, I called the nurse, turned it on again, and got up. Even now, if I turn off the machine, I cannot move, and I am out of breath.” (Participant 9)*

##### Theme 5.3. Fear of unexpected deep brain stimulation failure

Many participants expressed constant anxiety about unexpected device failure, which created an ongoing emotional burden and influenced both daily decision-making and healthcare-seeking behavior.

*“When I’m doing thermoelectric therapy or other tests, I’m very worried that the machine will suddenly break down, so I’m very worried about whether I should do the test or not.” (Participant 8)*

*“What if something happens and my device turns off? What will happen to me? Would I have to take several packs of medication again? I would be unable to move.” (Participant 12)*

These findings illustrate the profound psychological and physiological dependence on DBS among women with PD. The fear of immobility and breathlessness imposed a significant emotional burden, shaping daily activities, social participation, and access to medical care. These results underscore the need for healthcare providers to acknowledge and address DBS-related anxiety, offering tailored education, reassurance, and ongoing support.

### 3. Quantitative findings

After DBS implantation, participants’ baseline motor symptoms (UPDRS part III) improved significantly (39.32±10.40 before vs. 25.01±16.82 after, *p*=.038). The total UPDRS also improved significantly (69.82±16.01 before vs. 51.04±23.10 after, *p*=.018). Additionally, improvements were observed in axial symptoms (*p*=.003), PD stage progression (mean modified H&Y stage, *p*=.017), and a reduction in the number of patients with impulse control disorders (*p*=.025) (Table 2).

**Figure 1.**
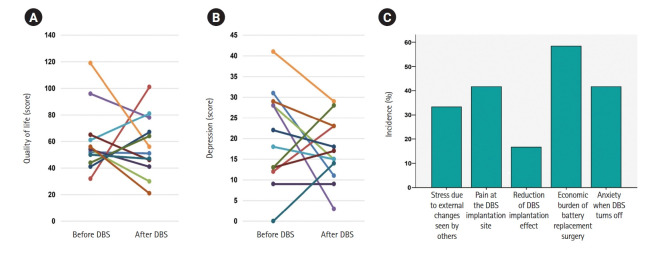
Changes after deep brain stimulation (DBS) implantation. (A) Changes in quality of life. (B) Changes in depression. (C) Proportion of unsatisfactory lived experiences after DBS implantation.

Changes in QoL and depression varied among participants, who were divided into those with improvement and those without (Figure 1). In QoL measures, the PDQ-39 subdomains of mobility, ADL, stigma, social support, and cognition showed improvement after surgery, but differences were not statistically significant. Conversely, emotional well-being (9.01±6.92 vs. 9.94±5.31, *p*=.610), communication (3.41±2.82 vs. 3.42±3.20, *p*=.944), and bodily discomfort (4.81±2.90 vs. 5.22±2.71, *p*=.721) worsened after surgery, though not significantly (Table 2). Depression scores decreased from 20.34±11.51 preoperatively to 17.12±7.70 postoperatively, but the difference was not significant (*p*=.450) (Table 2, Figure 1). No significant changes were observed in the use of SSRIs, LED, UPDRS parts I, II, and IV, motor fluctuations, or dyskinesia before and after DBS implantation (Table 2).

The DBS implantation period significantly differed between patients with improved PDQ-39 scores (n=7) and those without improvement (n=5) (*p*=.017). However, no significant baseline differences were found in demographic or clinical characteristics between the <5 years and ≥5 years DBS groups, except for the emotional well-being domain of QoL (*p*=.042) (Table 4). Nonsignificant variables included baseline age (*p*=.142), monthly income (*p*=.634), UPDRS scores (parts I–IV and total, *p*>.05), axial symptoms (*p*=.621), levodopa responsiveness (*p*=.871), LED (*p*=.953), total QoL score (*p*=.684), and depression (*p*>.999), indicating participant homogeneity across groups.

### 4. Synthesis of findings

The integration of qualitative and quantitative findings presented a coherent picture of the psychosocial challenges faced by women with PD after DBS implantation. Overall, the quantitative results complemented and reinforced the qualitative findings, particularly by quantifying the extent of psychosocial difficulties and QoL issues that emerged from patient narratives. While the qualitative phase revealed key themes such as emotional burden, social withdrawal, and economic stress, the quantitative results provided measurable confirmation of these experiences (Table 5). Although quantitative analyses showed no statistically significant improvements in overall QoL or depression, the qualitative data offered nuanced explanations for these outcomes. Interviews revealed persistent emotional burden, physical discomfort at the implantation site, and economic stressors, which limited perceived improvements in well-being despite objective relief of motor symptoms. Some participants did report subjective improvements in emotional well-being, which corresponded with upward trends in the QoL emotional well-being subdomain, though these did not reach statistical significance. This synthesis underscores the importance of integrating patient-reported experiences with objective data when evaluating high-technology interventions such as DBS. It highlights the necessity of interdisciplinary, patient-centered care strategies tailored to women with PD. Figure 1C illustrates the quantified frequency of the major themes identified through qualitative analysis. Following coding and theme extraction, the incidence of each theme was numerically summarized through frequency quantification to enhance clarity and facilitate comparisons across themes (Figure 1).

## Discussion

This study confirmed that although PD-associated motor symptoms (UPDRS part III) improved in Korean women with PD following DBS implantation, QoL and depression did not improve. A previous study examined the phenomenological experiences of DBS in 17 participants, including seven women with PD [20]. Although these women reported restored mobility—some even stating they would not have survived without the surgery—their sense of self fluctuated between deterioration and recovery after the procedure [20]. These findings align with the present study, suggesting that while DBS effectively alleviates motor symptoms, it does not necessarily translate into improved QoL or reduced depression.

Women with PD in this study described positive experiences after DBS surgery, particularly improvement in motor symptoms (UPDRS part III). This is consistent with prior research showing that both men and women experience comparable motor improvements after DBS [21]. However, it is notable that despite similar motor outcomes, women often report poorer physical functioning and socioemotional health-related QoL (HRQoL) compared with men [22]. This disparity suggests that DBS effectively addresses motor dysfunction but that additional support is needed to improve overall HRQoL in female patients. In this study, emotional well-being, communication, and bodily discomfort subdomains worsened after DBS compared to preoperative scores, although differences were not statistically significant. Postoperative difficulties—such as communication problems, pain or discomfort at the surgical site, and concerns about device maintenance—may contribute to physical discomfort and emotional distress in patients undergoing DBS [23,24].

The primary difference between patients with improved QoL and those without was the DBS implantation period. QoL tended not to improve in patients with longer implantation durations. Since baseline clinical characteristics did not significantly differ between patients with <5 years and ≥5 years of DBS, this stagnation cannot be attributed solely to implantation duration. Whether these results reflect the natural progression of PD or a decline in long-term DBS efficacy remains uncertain. Further research should examine potential confounding variables such as disease severity, medication adjustments, and psychological adaptation.

Participants also reported several dissatisfactory experiences with DBS implantation. Surgical sites on the chest and head were described as large, unsightly, and mildly painful, and patients feared device malfunction or shutdown. These experiences are similar to those of patients with implantable defibrillators [25]. However, few studies have examined the phenomenon from the perspective of patients with DBS [20,26-29].

Consistent with earlier studies addressing cosmetic outcomes of DBS battery insertion [30], this study suggests that, especially for women, consideration should be given to implanting the battery in the breast rather than beneath the clavicle. Such approaches could improve cosmetic outcomes and may serve as practical guidelines for future clinical decision-making.

Subjective sensations at surgical sites, including pain and abnormal perceptions, have been previously documented [31]. In a large quantitative study of 728 patients with movement disorders who underwent DBS, 1.1% reported discomfort at the electrode wire site in the neck, and 0.4% reported discomfort in the chest where the battery was implanted [31]. However, that analysis was limited because sex was not reported and subjective experiences were not explored. In the present study, the fear of device shutdown was consistent with experiences reported by female patients with pacemakers [24].

Financial burden also emerged as a major challenge for women in this study. As implantation duration increased, emotional well-being scores worsened. Yet previous research has not sufficiently addressed this issue, despite its growing relevance in Korea. Because costs such as battery replacement substantially affect patient well-being, further exploration of financial support mechanisms and policy-level interventions is warranted.

This study has limitations. Because it focused exclusively on female patients, including men in future research would enable a more comprehensive sex-based comparison of DBS efficacy, QoL outcomes, and psychological effects. Multicenter studies with larger patient populations are also needed to improve the generalizability of findings. This multimethod study showed that while women with PD who underwent DBS experienced nonsignificant reductions in depression and variable changes in QoL, some reported improvements in emotional well-being.

Despite motor symptom relief, participants expressed dissatisfaction due to persistent emotional distress, physical discomfort at implantation sites, anxiety related to device dependency, and particularly the financial burden of repeated battery replacement surgeries. Policy-based financial support—such as expanded public insurance coverage for battery replacement, subsidies for long-term device maintenance, and targeted programs for women with chronic neurological conditions—should be prioritized to alleviate economic strain and improve long-term outcomes.

## Figures and Tables

**Table 1.** Characteristics of the study participants (N=12)

Participant No.	Demographic characteristics					Disease-related characteristics				
	Age (year)	Marital status	Age at diagnosis (year)	Disease duration (year)	Age at DBS (year)	Implanted DBS duration (year)	Modified H&Y stage^†^ at medication initiation/discontinuation	Motor fluctuation/dyskinesia
1	66	Married	51	15.3	64	1	II/III	Yes/Yes
2	45	Married	32	12.8	40	5	II/III	Yes/Yes
3	61	Married	45	16.5	59	2	II/IV	Yes/None
4	59	Divorced	40	19. 3	54	5	II/II+	Yes/Yes
5	62	Widowed	32	30.4	44	18	III/IV	Yes/None
6	56	Divorced	40	16.3	51	5	II/II+	Yes/None
7	67	Married	45	22.3	56	11	III/IV	Yes/None
8	62	Married	46	16.3	55	7	II/II+	Yes/None
9	47	Married	30	17.3	37	9	II/III	Yes/Yes
10	40	Married	28	12.3	39	1	0/II+	Yes/None
11	63	Married	52	11.3	60	3	I/II+	Yes/Yes
12	62	Married	46	16.3	58	4	0/II	Yes/None

DBS: Deep brain stimulation surgery; H&Y: Hoehn and Yahr.

† Modified H&Y stage: 0, no signs of disease; I, unilateral involvement only; II, bilateral involvement without impairment of balance; II+, mild bilateral disease with recovery on pull test; III, mild or moderate bilateral disease, some posture instability, and physical independence; and IV, severe disability, still able to walk or stand unassisted.

**Table 2.** Characteristics of women with Parkinson disease who underwent DBS implantation (N=12)

Variable	Categories	n (%) or mean±SD (range)		*p*
		Pre-DBS	Post-DBS	
Current age (year)			57.51±8.80 (40–67)	
Age at onset (year)			40.32±8.31 (28–52)	
Age at DBS implantation (year)			51.42±9.11 (37–64)	
Implantation period of deep brain stimulation (year)			5.90±5.01 (1–18)	
Disease duration (year)			17.23±5.10 (11–30)	
Surgery location	Subthalamic nucleus		11 (91.7)	
	Globus pallidus interna		1 (8.3)	
Occupation	Housewife/none		11 (91.7)	
	Production/labor		1 (8.3)	
Underlying diseases	Diabetes mellitus		2 (50.0)	
	Hypertension		1 (25.0)	
	Hypercholesterolemia		1 (25.0)	
Total score of PDQ-39		60.32±24.20	56.91±22.60	.695
Mobility		22.80±8.11	21.01±11.13	.666
Activities of daily living		7.91±5.40	7.72±5.00	.858
Emotional well-being		9.01±6.92	9.94±5.31	.610
Stigma		6.31±3.31	4.21±4.23	.141
Social support		2.32±1.80	2.13±2.02	.573
Cognition		3.90±3.01	3.51±2.80	.642
Communication		3.41±2.82	3.42±3.20	.944
Bodily discomfort		4.81±2.90	5.22±2.71	.721
Beck Depression Inventory scale		20.34±11.51	17.12±7.70	.450
Prolonged use of SSRIs		2 (8.3)	2 (8.3)	>.999
UPDRS total	DBS on (medication off)	69.82±16.01	51.04±23.10	.018
	DBS off (medication off)	69.82±16.01	65.41±21.19	.583
UPDRS part Ⅰ	DBS on (medication off)	3.50±3.61	2.41±1.20	.721
	DBS off (medication off)	3.50±3.61	2.52±1.80	.593
UPDRS part Ⅱ	DBS on (medication off)	18.70±6.52	19.39±10.21	.372
	DBS off (medication off)	18.70±6.52	19.42±10.20	.755
UPDRS part Ⅲ	DBS on (medication off)	39.32±10.40	25.01±16.82	.038
	DBS off (medication off)	39.32±10.40	37.4±15.0	.790
UPDRS part IV	DBS on (medication off)	8.32±2.24	6.12±2.60	.056
	DBS off (medication off)	8.32±2.24	6.12±2.60	.056
Axial symptoms^†^	DBS on (medication off)	9.01±3.01	5.41±3.40	.003
	DBS off (medication off)	9.01±3.01	13.52±6.61	.016
Motor fluctuation	No	0 (0)	0 (0)	>.999
	Yes, but no discomfort	3 (25.0)	4 (33.3)	
	Yes, and discomfort	9 (75.0)	8 (66.7)	
Dyskinesia	No	5 (41.7)	7 (58.3)	.688
	Yes	7 (58.3)	5 (41.6)	
Off duration	No	0 (0)	0 (0)	.008
	≤25% of the day	9 (75.0)	1 (8.3)	
	≥26% of the day	3 (25.0)	11 (91.7)	
Modified Hoehn and Yahr stage		2.81±0.62	2.15±0.84	.017
Impulse control disorders (yes)		6 (50.0)	1 (8.3)	.025
Levodopa equivalent daily dose (mg/day)		1064.20±485.04	942.21±505.51	.695

DBS, Deep brain stimulation; PDQ-39, Parkinson’s Disease Questionnaire-39; SSRIs, selective serotonin reuptake inhibitors; UPDRS, Unified Parkinson’s Disease Rating Scale. Continuous variables were compared using the Mann-Whitney U-test and categorical variables using the chi-square test.

† UPDRS subscores for axial symptoms, defined as the total score of speech, neck rigidity, arising from a chair, posture, gait, and postural stability (UPDRS items 18, 22, and 27–30), were evaluated in the medication-off state.

**Table 3.** Theme clusters and subthemes

Theme cluster	Subthemes
Stress due to external changes seen by others	• Visible scars
	• Others’ reactions
Pain at the DBS implantation site	• Head pain
	• Chest pain
Reduction in DBS effectiveness	• Diminishing relief
	• Need for reprogramming
Economic burden of battery replacement surgery	• High surgery cost
	• Financial concerns
Anxiety when DBS turns off	• Fear of symptom return
	• Battery downtime worry

DBS, Deep brain stimulation.

**Table 4.** Comparison of the homogeneity of baseline characteristics according to DBS implantation period (N=12)

Variable	Categories	DBS implantation period, n (%) or mean±SD (range)		*p*
		<5 years (n=5)	≥5 years (n=7)	
Age at onset (year)		44.12±9.74	37.60±6.54	.142
Implanted period of DBS (year)		2.23±1.34	8.64±4.81	.017^*^
Disease duration (year)		14.32±2.34	19.32±5.72	.039^*^
Monthly income (US dollar)	<1,560	3 (25.0)	2 (16.7)	.634
	≥1,560	3 (25.0)	4 (33.3)	
Levodopa equivalent daily dose (mg/day)		1,010.34±337.41	1,102.71±592.72	.953
Improvement rate at levodopa before surgery (%)		75.13±24.72	73.45±18.03	.871
	UPDRS total	73.54±17.73	67.24±15.60	.234
	UPDRS part Ⅰ	3.82±4.40	3.32±3.23	.225
	UPDRS part Ⅱ	22.01±7.51	16.32±10.14	.624
	UPDRS part Ⅲ	40.32±12.03	38.64±16.86	.551
	UPDRS part IV	7.43±2.11	9.02±2.21	.863
	Axial symptoms^†^	9.51±3.21	8.62±3.02	.621
Modified Hoehn and Yahr stage		2.82±0.70	2.94±0.62	.599
Total score of PDQ-39		53.24±2.31	65.43±31.60	.684
	Mobility	20.84±6.10	24.14±9.52	.463
	Activities of daily living	8.01±4.72	7.90±6.24	.806
	Emotional well-being	4.23±3.70	12.40±6.81	.042^*^
	Stigma	6.82±2.61	5.90±3.83	.624
	Social support	2.23±1.34	2.30±2.14	>.999
	Cognition	2.80±1.91	4.71±3.53	.366
	Communication	4.20±3.30	2.91±2.54	.461
	Bodily discomfort	4.24±2.30	5.32±3.41	.620
Beck Depression Inventory Scale		19.42±14.01	21.04±10.62	>.999

DBS, Deep brain stimulation; PDQ-39, Parkinson’s Disease Questionnaire-39; UPDRS, Unified Parkinson’s Disease Rating Scale. Continuous variables were compared using the Mann-Whitney U-test and categorical variables using the chi-square test.

* *p*<.05.

† UPDRS subscores for axial symptoms, defined as the total score of speech, neck rigidity, arising from a chair, posture, gait, and postural stability (UPDRS items 18, 22, and 27–30), were evaluated in the medication-off state.

**Table 5.** Synthesis of quantitative and qualitative findings: depression, QoL, and unsatisfactory lived experiences after DBS implantation

Synthesis of qualitative and quantitative data	Qualitative data	Quantitative data
Economic burden negatively affects mental health and QoL	*“In the future, if the disease continues and there is no money, the cost of replacing the battery will be a burden to cover. Every time I change the battery, it costs a lot. I am concerned about where I can get financial assistance. Otherwise, I will have no choice but to die.” *(Participant 6)	•BDI score: mean 17.12–20.34 (indicating persistent mild to moderate depression, *p*>.05)
		•PDQ-39 total score: mean 56.91–60.32 (lower QoL, *p*>.05)
Persistence of QoL issues after DBS implantation	*“It has been more than 3 years since the operation and its effects are wearing off now. (After surgery) It was okay at first. The effect of the medicine was initially strong, but its effect has been decreasing with time. It is very difficult these days.” *(Participant 4)	•PDQ-39 total score change: no statistically significant improvement between <5 years and ≥5 years follow-up (*p*>.05)
Motor symptom improvement does not directly lead to mental health improvement	*“There has been a protrusion on the upper part of my chest, so I feel uncomfortable when lying on my side or exercising. *(Participant 10)	•BDI score change: no statistically significant reduction after DBS implantation (*p*>.05)
	*“I have never been to a public pool since I do not want others to see the surgical site. I am afraid I will be asked (touches her chest) what’s protruding, so I guess... I am afraid to think of it as a strange robot, so I never go to the public pool.” *(Participant 11)	

BDI, Beck Depression Inventory; DBS, deep brain stimulation; PDQ-39, Parkinson’s Disease Questionnaire; QoL, quality of life.
